# De novo assembly and characterization of leaf transcriptome for the development of functional molecular markers of the extremophile multipurpose tree species *Prosopis alba*

**DOI:** 10.1186/1471-2164-14-705

**Published:** 2013-10-14

**Authors:** Susana L Torales, Máximo Rivarola, María F Pomponio, Sergio Gonzalez, Cintia V Acuña, Paula Fernández, Diego L Lauenstein, Aníbal R Verga, H Esteban Hopp, Norma B Paniego, Susana N Marcucci Poltri

**Affiliations:** 1 Instituto de Recursos Biológicos, IRB, Instituto Nacional de Tecnología Agropecuaria (INTA Castelar), CC 25, Castelar B1712WAA, Argentina; 2 Instituto de Biotecnología, CICVyA, Instituto Nacional de Tecnología Agropecuaria (INTA Castelar), CC 25, Castelar B1712WAA, Argentina; 3 Instituto de Fisiología y Recursos Genéticos Vegetales (IFRGV), Centro de Investigaciones Agropecuarias (CIAP), Instituto Nacional de Tecnología Agropecuaria (INTA), Camino 60 Cuadras, km 5.5, X5020ICA, Córdoba, Argentina; 4 Facultad de Ciencias Exactas y Naturales, Universidad de Buenos Aires, Buenos Aires, Argentina; 5 CONICET, Buenos Aires, Argentina

**Keywords:** *Prosopis alba*, Fabaceae, Pyrosequencing, Transcriptome assembly, SSRs, SNPs, Functional annotation

## Abstract

**Background:**

*Prosopis alba* (Fabaceae) is an important native tree adapted to arid and semiarid regions of north-western Argentina which is of great value as multipurpose species. Despite its importance, the genomic resources currently available for the entire *Prosopis* genus are still limited. Here we describe the development of a leaf transcriptome and the identification of new molecular markers that could support functional genetic studies in natural and domesticated populations of this genus.

**Results:**

Next generation DNA pyrosequencing technology applied to *P. alba* transcripts produced a total of 1,103,231 raw reads with an average length of 421 bp. *De novo* assembling generated a set of 15,814 isotigs and 71,101 non-assembled sequences (singletons) with an average of 991 bp and 288 bp respectively. A total of 39,000 unique singletons were identified after clustering natural and artificial duplicates from pyrosequencing reads.

Regarding the non-redundant sequences or unigenes, 22,095 out of 54,814 were successfully annotated with Gene Ontology terms. Moreover, simple sequence repeats (SSRs) and single nucleotide polymorphisms (SNPs) were searched, resulting in 5,992 and 6,236 markers, respectively, throughout the genome. For the validation of the the predicted SSR markers, a subset of 87 SSRs selected through functional annotation evidence was successfully amplified from six DNA samples of seedlings. From this analysis, 11 of these 87 SSRs were identified as polymorphic. Additionally, another set of 123 nuclear polymorphic SSRs were determined in silico, of which 50% have the probability of being effectively polymorphic.

**Conclusions:**

This study generated a successful global analysis of the *P. alba* leaf transcriptome after bioinformatic and wet laboratory validations of RNA-Seq data.

The limited set of molecular markers currently available will be significantly increased with the thousands of new markers that were identified in this study. This information will strongly contribute to genomics resources for *P. alba* functional analysis and genetics. Finally, it will also potentially contribute to the development of population-based genome studies in the genera.

## Background

The genus *Prosopis* Linnaeus emend Burkart, a member of the subfamily Mimosoideae within the family Fabaceae, comprises 44 species divided into 5 sections: *Prosopis, Anonychium, Strombocarpa, Monilicarpa and Algarobia*[1]. This genus is spread around the world in arid and semiarid regions, including North and South America, North and Central Africa, Near East and the Caribbean region. The main center of diversity for *Prosopis* genus is located in Argentina [1] with 27 species. Of these species, 21 belonging to *Algarobia* section [2], which are distributed in the phytogeographic provinces of Chaco, Monte, and Espinal [3]. They cover over one million square kilometers, which represents approximately one third of the total country area [4].

One of the most important features of this genus is its natural capacity to produce fertile interspecific hybrids [5-7]. This generates a syngameon complex integrated by species and subspecies which form a continuum [8]. This complex includes six taxonomic species that play a significant role in Argentina: *P. alba, P. hassleri, P. nigra, P. ruscifolia, P. chilensis and P. flexuosa.*

The members of this complex develop deep roots that give these plants several advantaged. For instance, these deep roots reduce competition for water with herbaceous species, improve water balance of the system, provide nutrients to the subsurface layers and in some cases make the plants fairly independent of rainfall [9].

Their fruits are pods and may contain large amounts of sugar and protein which offer optimal energy for its use as fodder and for human consumption. They can also be used for firewood and charcoal, as well as for other products (honey, pollen, gums, etc.) [10]. Also, “algarrobos” can be an alternative of livestock-forestry systems [11].

Within this group of “algarrobos”, *P. alba* known as "white algarrobo" displays the widest geographical distribution. This species grows in areas under average annual precipitations of 500 to 1200 mm, which are summer dominant, with extreme temperatures between 48°C maximal absolute, up to −10°C absolute minimum [12]. *P. alba* comprises groups with different morphological characteristics, such as variations in leaves and fruits, and inhabits different ecological zones [13]. Also, these morphological groups have distinct adaptation mechanisms to drought stress [14].

In Argentina, this native species is mainly used for saw timber (wood flooring and furniture) and the whole wood consumed comes from the native forests in “Parque Chaqueño” (Argentina) [15].

Besides, all “algarrobos”, including *P. alba*, may play a role on the recovery of degraded ecosystem [16]; hence re-population with these species generates favourable conditions for natural recovery of the whole ecosystem.

Up to date, few genomic data exist on *Prosopis* genus. A total of 1,467 expressed sequence tag (EST) from *Prosopis juliflora* has been deposited in the NCBI EST database [17]. There are also a limited number of molecular markers published: six microsatellites isolated from *Prosopis chilensis*[18] and 12 from a bulk sample of American algarrobos introduced to Australia [19].

To the best of our knowledge, here we report the largest contribution to sequence information of *Prosopis* spp. generated through new generation sequencing technologies. The results from the assembly and functional annotation of *P. alba* leaf transcriptome are presented, along with SSR and SNP motif mining*.* Nuclear and chloroplast SSR and SNP were discriminated in the analysis. Finally, this work generated a collection of 11 nuclear-SSR primer pairs validated for its application to diversity studies in *P. alba* and another set of 123 nuclear polymorphic SSRs determined in silico, of which 50% have the probability of being effectively polymorphic. The overall workflow of the project is represented in the Additional file 1.

## Results and discussion

### Transcriptome sequencing and assembly

**Figure 1:**
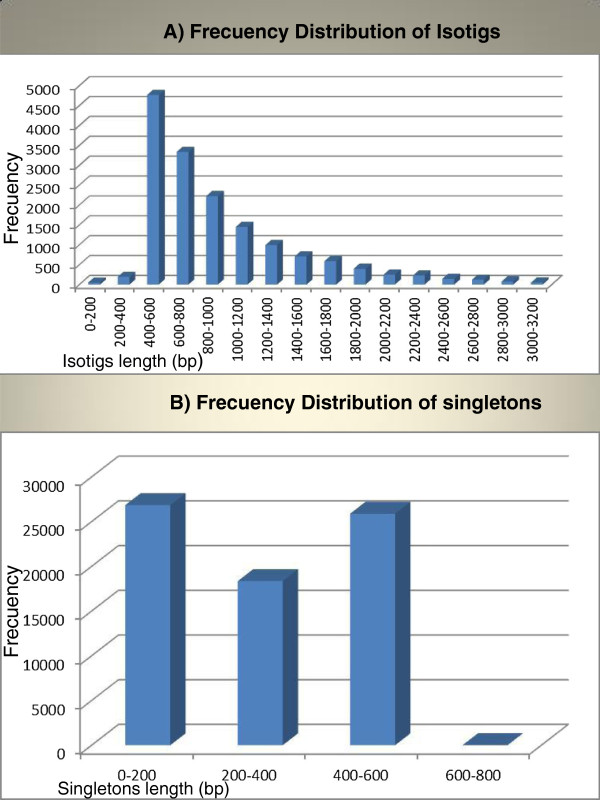
**Frequency distribution of isotigs (A) and singletons (B) lengths.** The histograms represent the number of isotig and singleton sequences in relation to their length.

An Rna-seq from a leaf bulk sample of three different individuals was performed using 454 GS FLX Titanium technology (Roche). The use of Rna-seq generated 464 Mb of sequence data from 1,103,231 reads with an average length of 421 bp, ranging from 21 to 692 bp. The sequences were subjected to filtering for adaptors, primer sequences and low-quality sequences. After this filtering, 39,711 reads were removed resulting in 1,063,520 high quality reads (96% of the first raw sequences). De novo transcriptome assembly was performed using Newbler Software v. 2.5 (Roche, IN, USA). With this assembly, 788,737 full length reads (74%) and 203,682 partial number of reads (19.3%) were assembled into 15,814 isotigs (equivalent to unique RNA transcripts) with an average length of 991 bp, ranging from 19 bp to 12,995 bp and N50 length of 1,124 bp (Figure 1A). In addition, the isotigs originated from the same contig-graph were grouped into 12,610 isogroups. These isogroups are equivalent to genomic loci (they contains the group of isotigs mapped to corresponding isogroups) with an average of 1.2 transcripts per locus, which potentially reflect multiple splicing variants. Most of the isogroups (86%) had only one isotig each. A total of 71,101 reads (6.6%) did not assemble into isotigs; therefore they were singletons. These singletons had an average length of 288 bp (Figure 1B) and, 32,991 of these singletons (85%) were shorter than 500 bp. All singletons were clustered using CD-HIT-454 algorithm to eliminate artificial duplicates. After clustering, we obtained 39,000 unique singletons longer than 200 bp. Then, all unigenes (whose length exceeded 200 bp (54,814 in total) were kept for further analysis (see Additional file 2, Table 1). The average length of *P. alba* isotigs was larger than those assembled in other native tree species *Nothofagus nervosa*, which had an average length of 765 bp [20]. The average length found in the present research was also larger than those of other non-model organisms ranging from 197 to 707 bp, [21-25]. This can be explained because of the use of the new 454 GS FLX Titanium (Roche) run that probably allowed us to obtain better and longer reads. Isotigs were integrated by different number of sub-contigs generated in the assembly process. These isotigs were integrated by 2 to 25 contigs with an average of 11 contigs assembled into each isotig. These results are similar to the average numbers obtained in the 454 *N. nervosa* transcriptome analyses (mean=9) [20] and larger than other 454 transcriptome analyses (mean=2.1), such as those of *Pinus contorta* and microalgae *Dunaliella tertiolecta*[23,24].

**Table 1:** **Transcriptome functional annotation summary of *P. alba***

**Number of sequences**			
	**Isotigs (15,814)**	**Singletons (39,000)**	**Combined (54,814)**
Viridiplantae DB			
Sequences with positive BLAST matches	14,664 (93%)	22,899 (59%)	37,563
Sequences annotated with Gene Ontology (GO) terms	10,107 (64%)	11,988 (31%)	22,095
Sequences without detectable BLAST matches	1,150 (7%)	16,101 (41%)	17,251
Sequences assigned to already known Enzyme Commission category	2,191 (14%)	2,347 (6%)	4,538

Number and percentages of 454 sequences in the assembled isotigs, singletons and total unigenes (combined) with significant matches against a Viridiplantae protein database.

### Functional annotation

For assigning putative functions to the *P. alba*´s transcriptome, BLASTX searches [26] were performed aligning the assembled sequences to the 1,958,459 protein sequences from a custom-made Viridiplantae database. A total of 14,664 isotigs and 22,899 singletons showed significant BLASTX matches (with an expectation value<1e-10) (Table 1). A higher percentage of isotigs (93%) than singletons (59%) had BLASTX hits, probably due to the good quality of isotigs (68% longer than 600 bp), short lengths of singletons and the high e-value cut-off applied. Previous reports on de novo transcriptome assemblies of eukaryotes described lower percentage of isotigs, ranging from 20 to 40%, such as those described for lanville fritillary butterfly, a coral larval, lodgepole pine and microalgae [21-24]. In total, 37,563 unique sequences had at least one BLAST hit in the search, while the 17,251 remaining sequences (i.e. 32%) (Table 1) were orphans. However, these orphan sequences may still be informative for identifying putative biological functions which may be considered as *P .alba* specific.

After the analyses of seven completely sequenced genomes, the average number of genes encoded in a plant nuclear genome was estimated in approximately 30 thousands [27]. Our annotated dataset with 12,610 isogroups, which can be used to estimate the number of gene locus, and 39,000 unique singletons most likely represent a good proportion of the *P. alba* gene catalogue.

**Figure 2:**
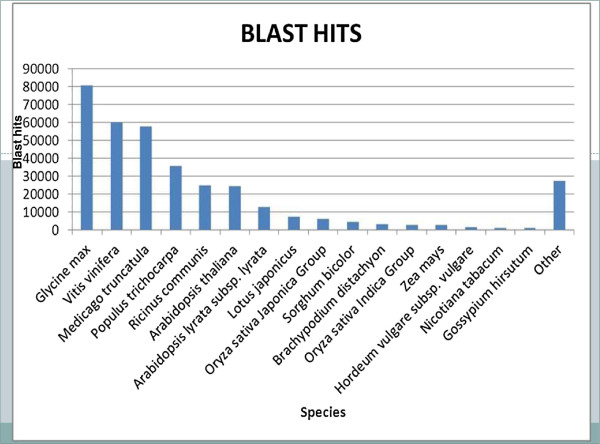
**Hit species distribution of BLASTX matches of *P. alba *unigenes.** Proportion of *P. alba* unigenes (isotigs and singletons) with similarity to sequences from Viridiplantae protein database.

BLASTX hits and top hits in terms of the total number of hits to all unigenes were mostly found with *Glycine max* (hits 80,668), *Vitis vinifera* and *Medicago truncatula* (Figure 2).

### Gene Ontology (GO) term annotation and metabolic pathway mapping

Using a full local installation of Blast2GO [28] and the InterProScan suite [29], we retrieved gene ontology (GO) terms and enzyme commission numbers (EC) for the *P. alba* unigenes (Additional file 2).

From the Blast2GO and InterProScan programs, a total of 43,389 GO terms were assigned to 22,095 unigenes (including 10,107 annotated isotigs and 11,988 annotated singletons). Among all the GO terms extracted, 14,422 (33%) belong to the Biological Process class, 19,077 (44%) fit the Molecular Function class and 9,890 (23%) belong to the Cellular Component class.

**Figure 3:**
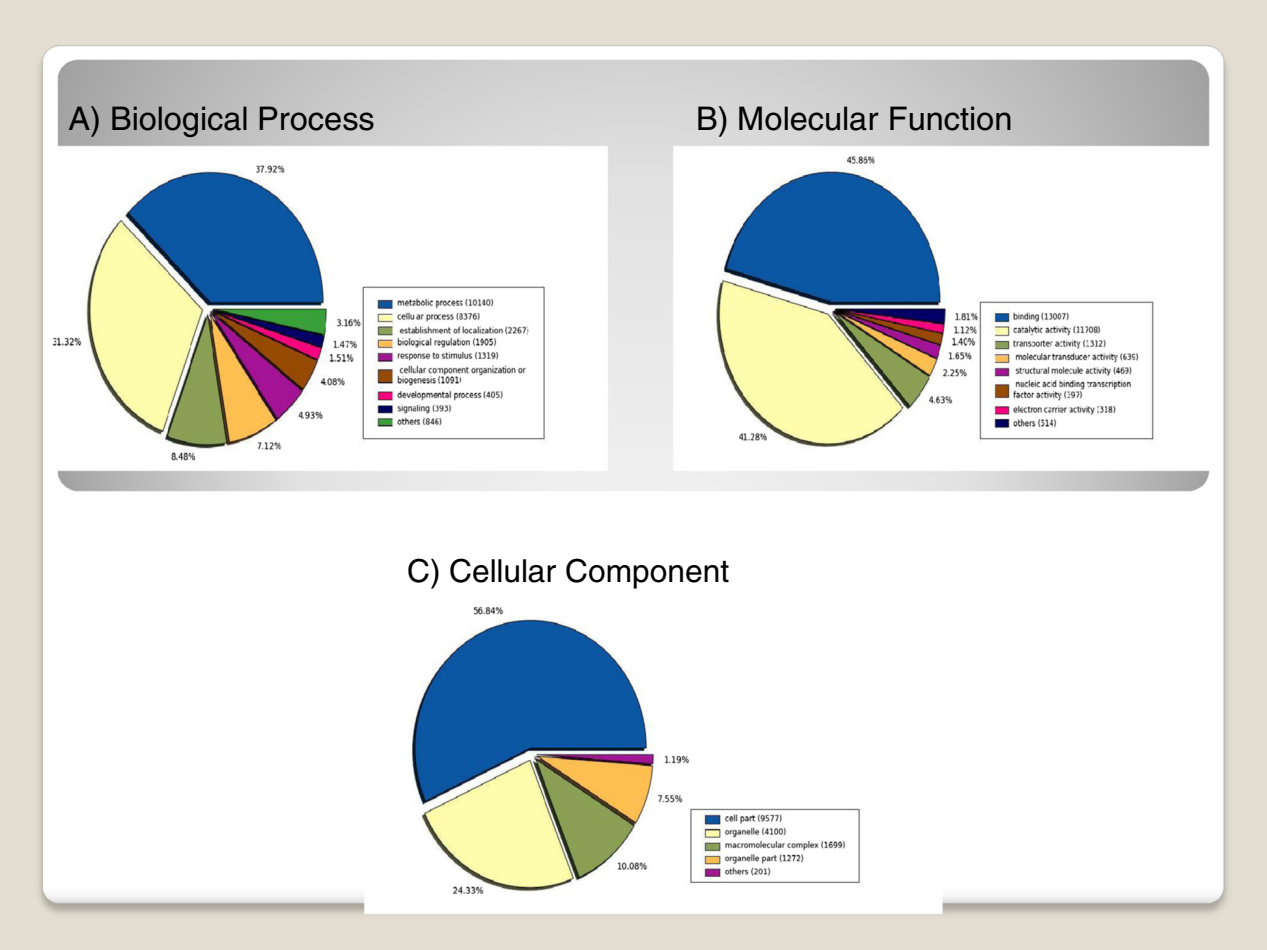
**Gene Ontology (GO) assignment of 37,563 *P. alba *unigenes in level 2.** The total number of unigenes annotated (22,095) for each main category are: 14,422 for “Biological Process” **(A)**, 19,077 for “Molecular Function” **(B)** and 9,890 for “Cellular Component” **(C)**.

The “Biological Process” (BP) GO category comprises different types of metabolic processes, which in turn are the most represented subcategories. Indeed, there are 10,140 sequences associated with metabolic processes (GO level 2) and 8,376 sequences related to cellular processes. These results may indicate that the analysed tissues were undergoing extensive metabolic activities [30]. These findings were expected, since the metabolic network in plants is very extensive compared to other organisms [31]. Within the sequences associated with biological processes, we found GO terms associated with primary and secondary metabolism. In this respect, the primary metabolites are known to be essential for plant survival and the secondary metabolites are described as playing roles in plant protection. Several genes involved in other important biological processes were also identified (Figure 3A). Among these genes, we can mention the ones associated with cellular processes, establishment of localization, biological regulation, biogenesis, developmental processes and signalling, to name a few. Another category worth mentioning is “response to stimulus” (BP category, level 2). We found 1,319 sequences in association with this category, which includes candidate genes involved in the resistance to biotic and abiotic stimulus.

In terms of molecular function, the top three GO terms found were related to the following categories: binding 13,007 (46%), catalytic activities, 11,708 (41%) and transporters 1,312 (5%) (Figure 3B).

A detailed analysis (level 2) at the cellular component category sorted all transcripts from *P. alba* into 5 groups. Of these groups, the most representative categories were: cell (9,577), organelle (4,100) and macromolecular complex component (1,699) (Figure 3C).

Of the 22,095 sequences annotated with GO terms, 4,538 were assigned with EC numbers (2,191 isotigs and 2,347 singletons) (Table 1).

**Figure 4:**
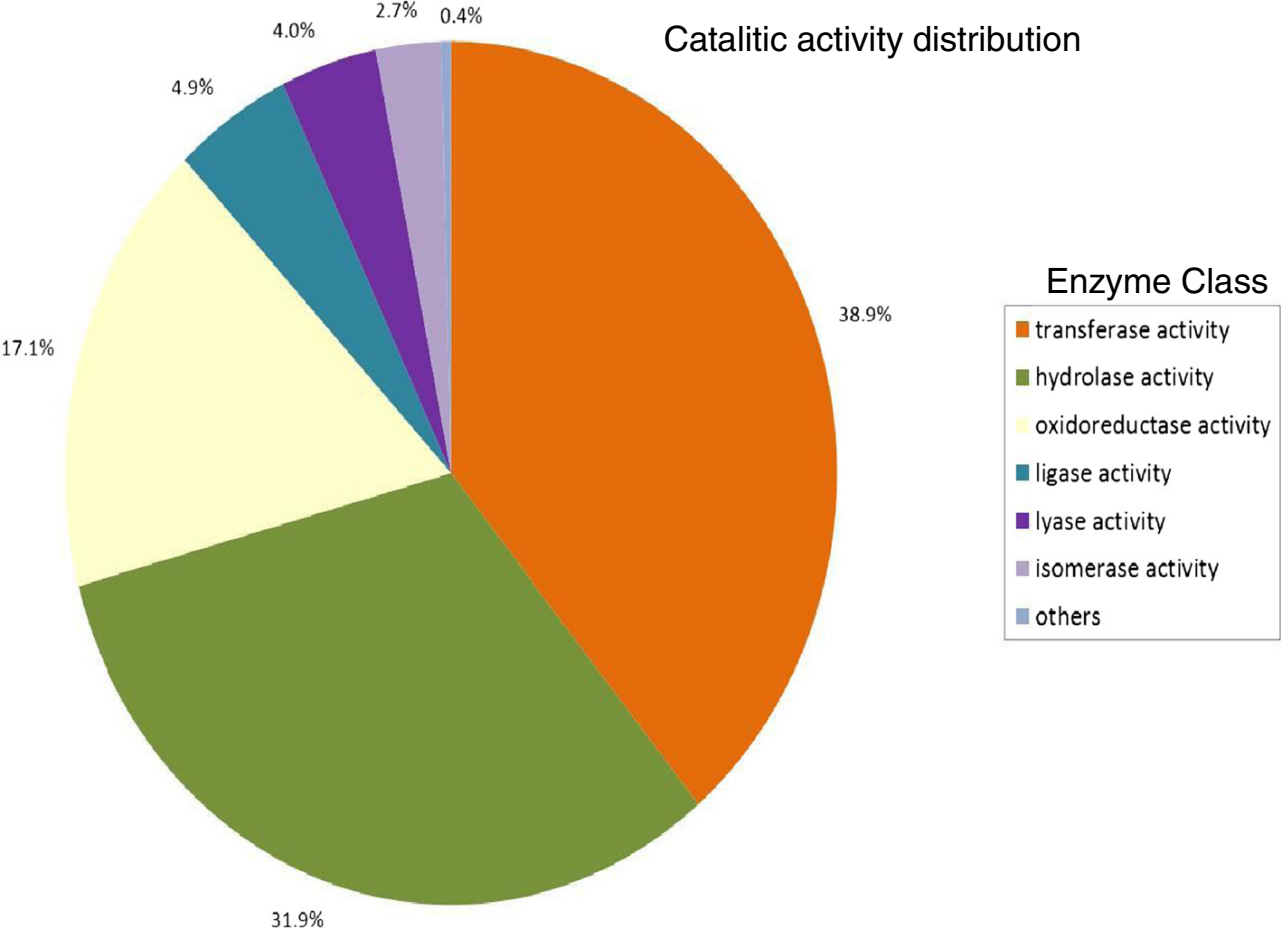
**Catalytic activity distribution in annotated *P. alba *** **unigenes.**

Figure 4 displays the most represented enzymes in all sequences: transferase activity (37%), hydrolase activity (35%) and oxidoreductase activity (13%). The large number of annotated enzymes within these three groups suggests the presence of genes associated to pathways of secondary metabolite synthesis [30,32,33].

To further enhance the annotation of the transcriptome dataset, all genes with GO terms were mapped to metabolic pathways using KEGG automatic annotation server (KAAS) [34]. From this analysis, 125 unique enzymes commission (EC) numbers were assigned to 22,095 genes, of which 31 unique enzymes were assigned to 50 metabolic pathways (Table 2) (Additional file 2).

**Table 2:** **Top metabolic pathways in *P. alba***

٭**Kegg metabolics patways**	**Number of unigenes**
Purine metabolism	485
Thiamine metabolism	476
Nitrogen metabolism	69
Oxidative phosphorylation	63
Phenylpropanoid biosynthesis	55
Lysine degradation	51
Starch and sucrose metabolism	42
Phenylalanine metabolism	36
Cyanoamino acid metabolism	31
Methane metabolism	28
Fatty acid biosynthesis	25
Other pathways	180

٭KEGG: Kyoto Encyclopedia of Genes and Genomes.

This table shows the KEGG metabolic pathways of plants that were well represented by unique sequences of *Prosopis alba*. The numbers of unigenes involved are described.

Regarding the analysis using KAAS, we found 485 transcripts involved in purine metabolism. This metabolic pathway is of fundamental importance in the growth and development of plants [35]. For instance, purine is involved in building blocks for nucleic acid synthesis and is also an energy source, as well as a precursor for the synthesis of primary products and secondary products [36,37]. Additionally, 476 genes associated with thiamine metabolism were detected. These genes are of particular interest to the *Prosopis* genus since the thiamine metabolism is involved in abiotic stress response through the Ca^2+^ salicylic acid and related signaling pathways [38].

When the metabolic pathways from *P. alba* was compared with other tree species (*N. nervosa*), we were able to observe 45 shared pathways. From these pathways, 8 were differential from *Prosopis* and 10 from *Nothofagus* (data not shown).

### Assessment of leaf transcriptome assembly

**Figure 5:**
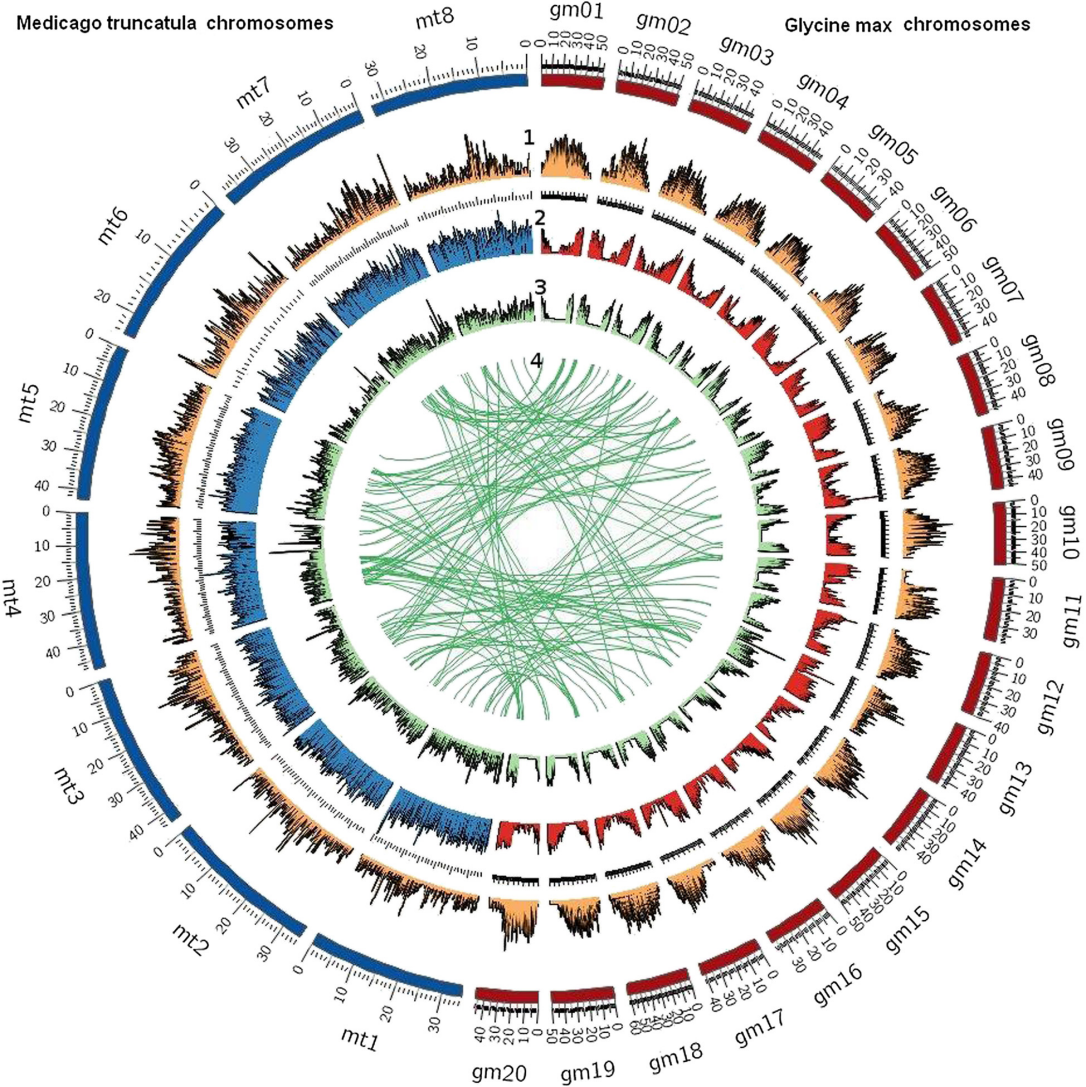
***P. alba *contigs/isotigs mapped to the *M. truncatula *and *G. max *genomes.** The 8 and 20 individual chromosomes of *M. truncatula* and *G. Max*, respectively, are shown in the outer circle (blue and red, respectively). Relative transposon and gene densities (by 100kb region) on each chromosome are successively displayed inward as follows: (i) Ring 1: transposon density in *M. truncatula* and *G. max* (orange), (ii) Ring 2: gene density in *M. truncatula* (blue) and *G. max* (red), (iii) Ring 3: *P. alba* gene density relative to gene densities of *M. truncatula* and *G. max* (green) (iv) Ring 4: sequences homologous to *G. max*, *P. alba* and *M. truncatula* altogether (central green lines).

For the assessment of the quality and completeness of the *P alba* transcriptome, a pair-wise reciprocal BLASTP was performed using the gene catalogues from *M. truncatula*, *G. max* and our *P alba* unigene dataset. All protein-coding loci were translated into their correct amino acid sequences and a BLASTP analysis was performed using the default parameters and an E-value cut-off of 10^10^. Sequences that were the reciprocal best hits among all 3 species were considered as the orthologue set and, taking this into account, 2500 *Prosopis* unigenes fell in this category. For the following analysis, the same strategy was followed but the comparison was carried out just between two species. The comparisons were performed with two legumes that have a complete genome assembly, *M. truncatula* and *G. max.* The results of these comparisons showed 4,872 and 4,703 unigenes from *P. alba* when compared to *M. truncatula* and *G. max* respectively. As test of the stringency of our strategy, we compared *M. truncatula* and *G. max* with each other and obtained a set of 15,219 orthologues as approximately expected with this strategy. In addition, to evaluate the distribution of all the *P. alba* isotigs along the eight chromosomes of *M. truncatula* and the 20 chromosomes of *G. max*, a protein-protein analysis was performed with “Promer”. This program translates all sequences into its six-open reading frames and makes an alignment [39]. The results of this analysis were plotted using a window size of 100kb through their genomic sequences (Figure 5). Transposon and gene densities in each species were distributed along all chromosomes of *M. truncatula* and *G. max*. As expected, in *G. max*, sequences of genes were most distributed in chromosome regions with low density of transposons. Genes belonging to *P. alba* distributed along all chromosomes in both species (Figure 5). However, sequences of genes were less represented in chromosome 6 of *M. truncatula*. This could also be seen in the central green lines (ring 4) that show the distribution of the 2,500 unigene homologous to *G. max*, *P. alba* and *M. truncatula* altogether.

Again, chromosome 6 of *M. truncatula* had fewer mRNA sequences homologous to *G. max* as well as with *P. alba*. When comparing *M. truncatula* genome with *Lotus japonica*[40], similar results were obtained. These findings demonstrate the lack of marker-based synteny with pea [41] and the abundance of nucleotide-binding site-Leu-rich repeat genes [42]. The unusual high proportion of heterochromatin in this chromosome as it was previously reported [43] may explain why we found less homologous mRNA sequences in chromosome 6 of *M. truncatula*.

### SNP detection

Single Nucleotide Polymorphisms (SNPs) were identified through the analysis of the multiple alignments produced during the assembly process. The criterion for this analysis was reducing the probability of false positive identification (see Materials and Methods).

The analysis of 15,814 isotigs resulted in the identification of 7,134 putative SNPs. After applying filters, we obtained a total of 6,236 high confidence SNPs from 1,834 isotigs (average = 3.4 SNPs per isotig). Of these SNPs, 70 belonged to 14 chloroplast isotig and 6 contig sequences (see Additional file 3). The 15,814 isotigs that were mined for SNPs identification comprised 15,665 KB of *P. alba* transcriptome, with 1 SNP per 2,512 bases on average. The SNPs density in “algarrobo” was similar to that found in *Capsicum annuum* trancriptome (1 SNP per 2,253 bases) [44]. In both cases the criteria to identified SNPs and the number of individual analysed were analogous. However, in oak [45] and in *Eucalyptus grandis*[46] the SNP frequency was much higher than in *P. alba,* (1 SNP every 471 bp and 1 SNP every 192 bp, respectively). These differences can mainly be attributed to the number of individuals that were sequenced in the other forest species (21 individuals of oaks and more than 200 individuals of *Eucalyptus grandis*). Within the identified SNPs, transitions (65%) were far more frequent than transversions (35%) (Table 3). A similar number of A/G and C/T transitions together with equivalent values of the four transversion categories (A/T, A/C, G /T, C/G) were found. These results are in accordance with the findings described in *Cucurbita pepo* SNPs [30].

**Table 3:** Single nucleotide polymorphism (SNPs) statistics

**SNPs**	**Number**	**SNPs**	**Number**
**Transitions**		**Transversions**	
A<−>G	2,076 (51%)	A<−>C	548 (25%)
C<−>T	2,004 (49%)	C<−>G	610 (28%)
		G<−>T	570 (26%)
		A<−>T	428 (20%)
Total	4,080 (65%)	Total	2,156 (35%)

Class and number of transitions and transversions are shown for putative high quality single nucleotide polymorphism (SNPs) identified in *P. alba* transcriptome.

### Single sequence repeats (SSRs) detection

Using the SSR webserver from the Genome Database for Rosaceae (GDR), we identified and characterized several SSRs (microsatellites) motifs as potential molecular markers in the *Prosopis* putative unigenes collection generated in this work.

The criterion used for the SSR selection was based on the minimum number of repeats (see Materials and Methods). These settings resulted in the identification of 5,956 nuclear SSRs within 54,768 unigenes, i.e. SSR frequency of 11% taking into account multiple repeat occurrences in a same unique locus. This frequency was comparable to that reported in *Nothofagus* (15%) [20] and lower than in oak (19 and 24%) [45,47]. A total of 4,990 (9%) nuclear unigenes contained at least one SSR, suggesting that they are distributed throughout the whole leaf transcriptome. Additionally, 4,593 SSRs (77 %) had sufficient flanking sequences to allow the design of appropriate unique primers to generate PCR products within the range of 100 to 300 bp. Detailed information of the SSRs that were discovered in this research is described in Additional file 3.

**Figure 6:**
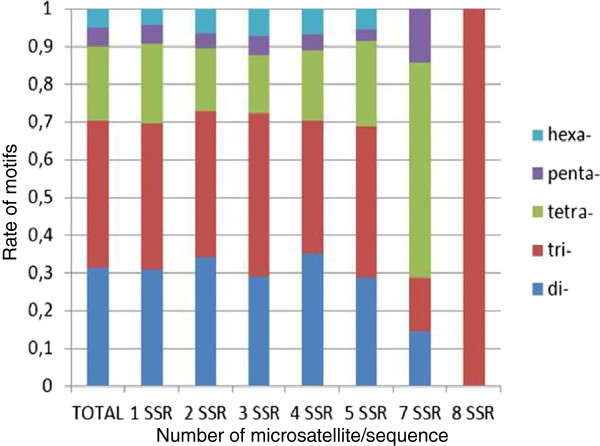
**Frequencies of SSRs in *P. alba *unigenes.** Frequencies of di- tri- tetra- , penta- and hexa nucleotide SSRs in unigenes containing one to eight SSRs per locus. Of the sequences, 4294 had one SSR, 602 contained two, 116 had three, 18 had four, six had five SSRs and one sequence showed 8 SSRs.

As expected, the most frequent type of microsatellite corresponded to trimeric repeats (41%), while much lower frequencies were found for dimeric motifs (29%), tetra- (20%), penta- (5%) and hexanucleotide repeats (5%) (Figure 6). Similar results were found in *Nothofagus* and oak [20,45].

Seventy two percent of the sequences had only one SSR (72%) and 20 % had two. Of the unique SSR, 44% were of trimeric motif followed by 28% of di- and 19% of tetranucleotide motifs. The SSRs were highly distributed over the sequences; which provides a useful tool for different genetic studies (Figure 6).

The topography of SSR distribution was analyzed for the presence of SSR within predicted UTRs and coding sequence regions (See Materials and Methods). About 44% of the SSR sequences were inside ORF sequences, being most of them trinucleotide and hexanucleotides repeats (58%). In the UTRs, the dinucleotides motifs were more frequent (35%) comparable to those reported in other trees such as oak (27%) [45], *Nothofagus* (40%) [20] and pines (65-75%) [48].

Eighty one percent of the repeated sequences found in ORF had a combination of length motif and repeat number multiple of three, (i.e., (TC)_9_, (GA)_9_, (CTCC)_3_, (CGCCC)_3_) which did not modify the reading frame, (i.e. nine motifs of two nucleotides =18 bp = 6 codons). This could be explained on the basis of the selective disadvantage of non-trimeric SSR variants in coding regions, possibly causing frame-shift mutations [49].

Finally, we also identified SSRs belonging to gene families associated with the production of cellulose, the lignin biosynthetic pathway [50] and with transcription factors. As for the production of cellulose, we detected genes such as cellulose synthases (CesA), glycosyl transferases and sucrose synthase. The identified genes related to the lignin biosynthetic pathway [50] were cinnamoyl alcohol dehydrogenase as well as cinnamoyl reductase-like protein. From the genes associated with transcription factors, zinc finger proteins and some anti-oxidants (for example, gdp-mannose pyrophosphorylase) were identified. Stress related sequences such as heat shock proteins and zeaxanthin epoxidase were also identified in the reference *P. alba* transcriptome, Interestingly, zeaxanthin epoxidase is a precursor of abscisic acid (ABA) that is involved in response to abiotic stress, including tolerance to heavy metals.

### Validation of the predicted microsatellite markers

Eighty seven microsatellites were selected according to their sequence length, GC content and functional annotation. As for functional annotation, we selected those related to the following categories: stress, calcium metabolism, peroxidases, myb and zinc finger proteins, among other putative functions (51% were located in predicted ORFs). The 87 loci were tested for PCR amplification in six individuals*.* All of them (100%) were effectively amplified validating the quality of the assembly and the utility of the SSRs herein identified. Similar results were obtained in *Nothofagus* by applying the same strategy for the assembly and the in silico searches for SSRs. Similar research carried out using Illumina sequencing technology in sesame showed that about 90% primer pairs successfully amplified DNA fragments [51]. However, the rate of SSR validation was lower (64.9%) when the marker mining was done using EST produced by Sanger technology [52], possibly because of the low-quality of the EST sequences. The lower rate could also be explained by the use of primer sequences derived from chimerical cDNA clones.

About 15% (11 SSR) of the tested *Prosopis* SSRs were polymorphic and showed at least one individual differing in allelic composition, belonging to 9 different loci positions ie: three SSRs belonged to the same isotig 00930. This relative low percentage of polymorphic loci could be due to the small sample size tested (six seedlings), the selected target loci (focused principally in abiotic stress) and their presence in predicted ORF. The percentage was slightly lower than that of a similar study in *Nothofagus* (20%). Furthermore, even higher percentages of polymorphism were identified in other reports: 30% in *Phoenix dactylifera L* date palm (assessed in 12 cultivars) [53], 46% in *E. globulus* (evaluated in 8 samples) [52] and 80% in sesame (assayed in 24 samples) [51]. Five of the polymorphic SSRs found in this work were located within predicted ORF and showed repeat motifs multiple of three (Table 4), therefore maintaining the coding region in frame [49].

**Table 4:** **Polymorphic SSR primer pairs derived from *P. alba *** **unigenes**

**Primer name**	**Marker ID name gene bank accession no.**	**Motif**	**ORF**	**Primer sequence 5´-3´**	**Amplicon Lenght**	**Seq description**
I-P00930b	isotig00930b	acc (5)	Y	F:GCAACAGCACTGCTTCAAA	260-268	Zinc finger protein magpie-like
				R:AAAATAGCGCCATAGTTTGCTC		
I-P00930c	isotig00930c	gtc (4)	Y	F:TATGGCGCTATTTTTGGAGG	236-240	Zinc finger protein magpie-like
				R: TCATGCTCCTCACAATCTGC		
I-P00930d	isotig00930d	aac (6)	Y	F:TCGAGATTTTCTTGGGGTTG	176-178	Zinc finger protein magpie-like
				R: AAATTCCCTCCTCCTCCAAA		
I-P03211	isotig03211a	aat (4)	Y	F: TTGCTTCAGAAAGCTGCTCA	190-198	Uncharacterized protein loc100815794
				R: AACCCTCGAAGATGATGGTG		
I-P03325a	isotig03325a	ca (5)	N	F: CGTGCATGAATGTCACAGAC	226-230	Peroxidase
				R:AGGGTGAGATCAGAAGGCAA		
I-P06286b	isotig06286b	tc (5)	N	F:TGACAACCCATCTTCTTCTTCA	206-216	Myb transcription factor myb138
				R:ATTTGCACAAGGGTAAAGATGG		
I-P06639	isotig06639a	at (5)	N	F: CATCCCGTTCAAGTCCAAGT	226-230	Aquaporin pip11
				R: AGCCCCCTTCCAACTTCTAA		
I-P07653	isotig07653a	gtt (4)	Y	F: AGTGATGATTCGGATCCTGG	216-220	zf-hd homeobox protein
				R:GAGAGACGAGGACTTGGTGC		
I-P10500	Isotig10500a	ttc (6)	N	F: CTCCGACAGATTCAGCATCA	260-275	Pentatricopeptide repeat-containing protein
				R: TTCTTTCAAACTCGCCATCA		
S-P1DKSFA	GR7D2IN01DKSFA	ttta (3)	N	F: GTTTACCCATTGCAGGTCGT	162-168	Calcium-binding mitochondrial carrier protein s −1
				R: CCCCATATGCAGAATCACCT		
S-P1EPIV2	GR7D2IN01EPIV2	taa (4)	N	F: TAAGCATTCATAGCCAGCCC	290-298	Peroxidase 73
				R: GACCAGGTCCTGTTTACCGA		

The included data are: primer names, marker ID names, motif and number of repeats, position in ORF, forward and reverse primer sequences (5´3´), amplicon length (bp), BLASTX similarity matches (Putative Function).

### Polymorphic SSR predicted

From the 2,352 nuclear SSRs detected in contigs/isotigs (which means they have several reads that allow to determine putative polymorphism), a total of 1,995 (85%) had defined primers for PCR reactions, belonging to 1,622 different isogroups (unigenes).

In order to predict nuclear polymorphic SSRs, we carried out in silico PCR for each of the sequences from the different contigs/isotigs. For this purpose, all 1,063,520 high quality reads were used. To achieve a higher success rate, another set of primers was also were designed closer to the SSR motifs in order to capture short length reads included in contigs.

At least 123 nuclear polymorphic SSRs were detected by PCR in silico; which only includes isotigs integrated by three or more reads and whose product size generated by in silico PCR differed in at least two base length (Additional file 3). An apparent underestimation of nuclear polymorphisms in silico was observed when considering that from the 9 clear polymorphic SSRs coming from isotigs (Table 4), of which, only two of them (22%) resulted polymorphic under the criteria used in silico. However, from the 87 SSRs that were amplified in vitro, 69 belonged to isotigs and only six had enough reads to be considered in this in silico analysis; which resulted in only three effectively polymorphic SSRs in silico (50%). Therefore, it can be predicted that 52 SSRs out of the 123 new SSRs that were detected in silico will be effectively polymorphic in vitro. This result could be an interesting survey of potential useful SSRs and could contribute significantly to the SSRs available in other reports [18,19].

A total of 135 GO terms were allocated to the 116 nuclear isogroups containing the polymorphic SSRs that were identified in silico in this research. They were assigned under the categories “Biological Process” (39 terms), “Molecular Function” (55 terms), and “Cellular Component” (41 terms). The most represented subcategories assigned under “Biological Process” at third-level terms were: “primary metabolic process” (16.4%), “cellular metabolic process” (15.6%) and “macromolecule metabolic process” (14%). In addition, many of the terms that classified as “Molecular Function” were represented by genes in the following subcategories: “hydrolase activity” (20%), “nucleic binding” (18%), “ion binding” (16%) and “protein binding” (13%). In addition, seven metabolic pathways were represented by at least one sequence, with its corresponding EC number. This makes these functional markers especially useful for population and evolution analyses of *P. alb*a.

### Chloroplasts mining

We detected 44,079 chloroplast reads through alignment analyses to related chloroplast (cp) sequences. After an alignment analysis with the legume *Vigna radiata* chloroplast genome, 56 contigs composed of 59,040 bp were generated, spanning a total of 129,208 bp that belong to the *Prosopis* cp genome. The chloroplast reads of *P. alba* with 59,040 bp represented ~40 % of the total cp genome of *V. radiata* (151,271 bp) [54]. There were 55 intra scaffold gaps in *P. alba* cp genome with a mean sequence gap size of 1252 bp.

A total of 14 isogroups harboring 36 SSRs were also found: 18 with designed primers, 17 with different BLASTX hits related to chloroplast metabolism (oxidoreductase, ribosomal, etc.) and four polymorphic in silico (Additional file 3). Chloroplast SSRs were previously described in several plants such as *Pinus radiata*, *Oryza sativa, Nicotiana*[55-57]. More recently several other organisms were also characterized for these SSRs: *Eucalyptus globulus, G. max, V. radiata, M. truncatula, V. vinifera* among others. All of these Chloroplast SSRs have been deposited in a data base http://www.mcr.org.in/chloromitossrdb/[58]. Also, in rice around 4.5% of the chloroplast genome has been covered by SSRs [59].

## Methods

### RNA preparation and cDNA library synthesis

Total RNA was extracted from leaves of seedling collected from natural populations of *P.alba* from different provinces of Argentina: population 1 from Campo Durán (province of Salta), population 2 from Isla Cuba (province of Formosa) and population 3 from Chañar Bajada (province of Santiago del Estero)

The RNA extraction method used in this research is the one described by Chang el al, (1993) [60]. Briefly, one gram of fresh tissue was ground to a fine powder under liquid nitrogen. Then, after two extractions with chloroform, the RNA was precipitated with LiCl_2_, extracted again with chloroform and finally precipitated with ethanol. The resultant RNA was resuspended in 50 μl of DEPC treated water. The RNA was quantified using a Nanodrop 1,000 spectrophotometer and its quality was measured with a 2,100 Bioanalyzer (Agilent Technologies Inc.). Then, it was subjected to purification using the Poly (A) Purist kit (Ambion) and its quality was once more assessed with the 2,100 Bioanalyzer. cDNA was synthesized using cDNA Kit (Roche) and used to construct a shotgun library for pyrosequencing technology (Roche). The *Prosopis* cDNA library was subjected to a 1/3 of plate production run on the 454-GS-FLX sequencing instrument. This run was conducted at INDEAR (Rosario Biotechnology Institute, Rosario, Argentina).

### Transcript assembly and analysis

The sequences were subjected to filtering for adaptors, primer sequences and low-quality sequences After removing the low quality sequences, the curated raw 454 read sequences were assembled into contigs, isotigs and isogroups using Newbler Assembler software 2.5p1 (Roche, IN, USA). The reads identified like singletons (i.e., reads not assembled into isotigs) after assembly were subjected to CD-HIT-454 clustering algorithm at 95% identity cutoff, which eliminates redundant sequences [61].

BLASTX (e-value cut off ≤ 10e-10) searches were performed against a Viridiplantae protein database first. Then, the sequences with no hits were used to perform a successive BLASTX against the NCBI nr protein database in order to make an assessment of the putative identities of the sequences. Unigenes (>200 bp) were deposited at the National Centre for Biotechnology Information (NCBI) Transcriptome Shotgun Assembly (TSA) Database under BioProject: 218545 TSA- SUB336788.

Annotation and mapping routines were run with BLAST2GO [28], which assigns Gene Ontology ([62], http://www.geneontology.org) annotation, KEGG maps (Kyoto Encyclopedia of Genes and Genomes, KASS) and an enzyme classification number (EC number) using a combination of similarity searches and statistical analysis [34]. In addition to BLAST2GO, the full suite of InterProScan was ran with default parameters. InterProScan combines different protein signature recognition methods native to the InterPro member databases into one resource that searches for the corresponding InterPro and GO annotations.

Chloroplast assembly analysis was carried out using AMOScmp [63]. To search for chloroplast sequences, BLASTN and TBLASTX (BLASTN e-50 TBLASTX e-10) were performed. The analysis was based on similarity with and without translation to 109 chloroplast genomes (ftp://ftp.ncbi.nlm.nih.gov/genomes/Chloroplasts/plastids/).

### Comparative genomics

Circos software tool [64] was used to visualize *P. alba* sequences with *M. truncatula* and *G. max*´s genomes, through circular concentric ideograms layout to facilitate the display data as scatter, line and histogram plots for each different sample. Promer analysis was performed and filtered by using a window size of 100kb through their genome sequences. Homologous sequences for the three species were determined when reciprocal TBLASTX best hits were found for the three “genes” tested.

### SNP identification

In order to perform matching, alignment of DNA sequences and searching for putative SNPs, the SSAHAsnp Program (Sequence Search and Alignment by Hashing Algorithm) was used [65]. The criterion designed to reduce the probability of false positive identification was that the minority allele (the second most common nucleic allele) should be found in at least 4 sequences and that at least the 10% of reads had an SNP from total coverage, which should be at least 8x.

### SSR identification

In order to identify SSRs for all possible combinations of dinucleotide, trinucleotide, tetranucleotide and pentanucleotide repeats, we performed a run using the SSR webserver (GDR) (http://www.rosaceae.org/bio/content?title=&url=/cgi-bin/gdr/gdr_ssr). This webserver uses the GETORF algorithm (EMBOSS Package) and selects the longest ORF as the putative coding region. This webserver also uses Primer 3 (v.0.4.0) [66] to design primer pairs. The criteria used for the SSR selection based on the minimum number of repeats was as follows: five for dinucleotide, four for trinucleotide, three for tetranucleotide and three for penta and hexanucleotide motives.

The presence of expressed repetitive DNA was revealed using the BLASTN (e-value cut off ≤10e-10) searches against all Viridiplantae Repbase.

### SSR validation

For validation of SSR primers, total DNA was extracted from young leaves of six *Prosopis alba* seedlings from three native populations described previously (two for each one). For DNA extraction, the Dneasy Plant mini kit (Quiagen) was used following the manufacturer´s instructions. Regular primers were synthesized (AlphaDNA, Montreal, CA, USA) and used for PCR (polymerase chain reaction) amplification. PCR reactions consisted of 20 ng of total DNA, 0.25 μM of each primer, 3 mM of MgCl_2_, 0.2 mM of each dNTP, 1X of PCR buffer and 1 U of Platinum Taq polymerase (Invitrogen). All PCRs were performed with the following conditions: a denaturation step of 2 min at 94°C, a regular touchdown PCR ranging from 60°C to 50°C with 28 cycles at the touchdown temperature of 50°C (45 s at 92°C, 45 s at 50°C and 45 s at 72°C). The final extension step was of 10 min at 72°C and then the temperature conditions were adjusted for each particular microsatellite. Samples were mixed with denaturing loading buffer, incubated for 5 min at 95°C, and separated on a 6% polyacrylamide gel. Amplification products were stained using the DNA silver staining procedure of Promega (USA) following the manufacturer’s instructions. Details of primers sequences, SSR location and amplicon sizes are described in Table 4.

## Conclusions

The *P.alba* transcriptome database obtained and characterized here represents a major contribution for *Prosopis sp.* genomics and genetics. It will be useful for discovering genes of interest and genetic markers, which could allow to investigate functional diversity in natural populations. These tools will also lead to conduct comparative genomics studies with other *Prosopis* species taking advantage of their remarkable ecophysiological differences. This work highlights the utility of transcriptome high performance sequencing as a fast and cost effective way for obtaining rapid information on the coding of genetic variation in *Prosopis* genus. This study allowed us to: (i) obtain 1,103,231 transcript raw reads and 54,814 unigene sequences from *P.alba*, (ii) identify putative function in 37,563 unigenes for the genus, (iii) identify 700 putative stress-response genes, (iv) discover 4,593 genomic SSRs with designed primers, validate 87 and detect 11 polymorphic SSRs, several of them related to response to stress, (v) identify probably 52 effectively polymorphic after in silico analysis, and (vi) identify 6,158 higher confidence nuclear SNPs, some of them related to the production of cellulose, together with the lignin biosynthetic pathway and with stress, among others.

## Competing interests

The authors declare that they have no competing interests.

## Authors’ contributions

SLT organized the research, provided funds, contributed to RNA extraction, data analysis and wrote this manuscript. MR coordinated and carried out bioinformatics analyses and contributed to the manuscript, MFP contributed to RNA extraction and carried out SSR validation. SG carried out the bioinformatics analyses. CVA contributed to the analyses involving BLAST, SSR characterization and with the manuscript revision. PF contributed to RNA extraction. DLL contributed to write the project and manuscript, ARV provided the biological material for transcriptome sequencing and contributed to manuscript revision. HEH conceived this study and contributed to the revision of the manuscript. NBP provided funding, participated in the design of the bioinformatics study and reviewed the manuscript. SNMP provided funding, contributed to research design, data analysis, contributed to the manuscript and its review. All authors approved the final manuscript.

## Supplementary Material

Additional file 1 **Schematic representation of the overall sequencing and annotation workflow of *Prosopis alba *transcriptome.** The steps and sets of sequences involved in transcriptome sequencing, assembly of reads, annotation using protein databases, the statistical thresholds, filters, genetic marker discovery and characterization. Click here for file

Additional file 2 **Annotation.** This table provides information on the annotation of isotigs and singletons, GO information and the enzymes putatively encoded by the RNA sequences, based on homology prediction and their associated pathways. This includes KEGG maps, enzyme names, and sequences ID. Click here for file

Additional file 3 **In silico SSRs and SNPs derived from *Prosopis alba *leaf transcriptome.** The data describe the 5,996 SSR and 6236 SNPs. Sheet SSR: Included are unigenes names, Isogroup, marker ID, Sequence Lenght (bp), SSR Polym: (In silico "IS" or "IS(2)"=two sequences in pcr in silico, PCR Amplification: "POLYM", "COMPLEX PATTERN", " MONOM", >mw=molecular weight out of range) SSR description: # SSRs per seq, repeat lenght, motif, # Repeats, SSR position (start, stop) ORF definition (start, stop, SSR in ORF) primers description: sequence of forward and reverse primers, expected product size (bp), similarity matches, E value, similarity mean, #GO, GO terms, Enzimes codes and their chloroplast belonging. Sheet SNP: Included are unigenes names, Isogroup, marker ID, SNP position, SNP, # of mapping reads with SNP, # total reads coverage on the SNP position, # of mapping SNP reads vs consensus, % of reads with SNP, similarity matches, E value, similarity mean, #GO, GO terms, Enzimes codes , chloroplast belonging. Click here for file
