# Supplementary figures and images for: De novo assembly and characterization of leaf transcriptome for the development of functional molecular markers of the extremophile multipurpose tree species Prosopis alba
Source: BMC Genomics. 2013 Oct 14;14:705. doi: 10.1186/1471-2164-14-705 (PMC4008253; doi:10.1186/1471-2164-14-705)

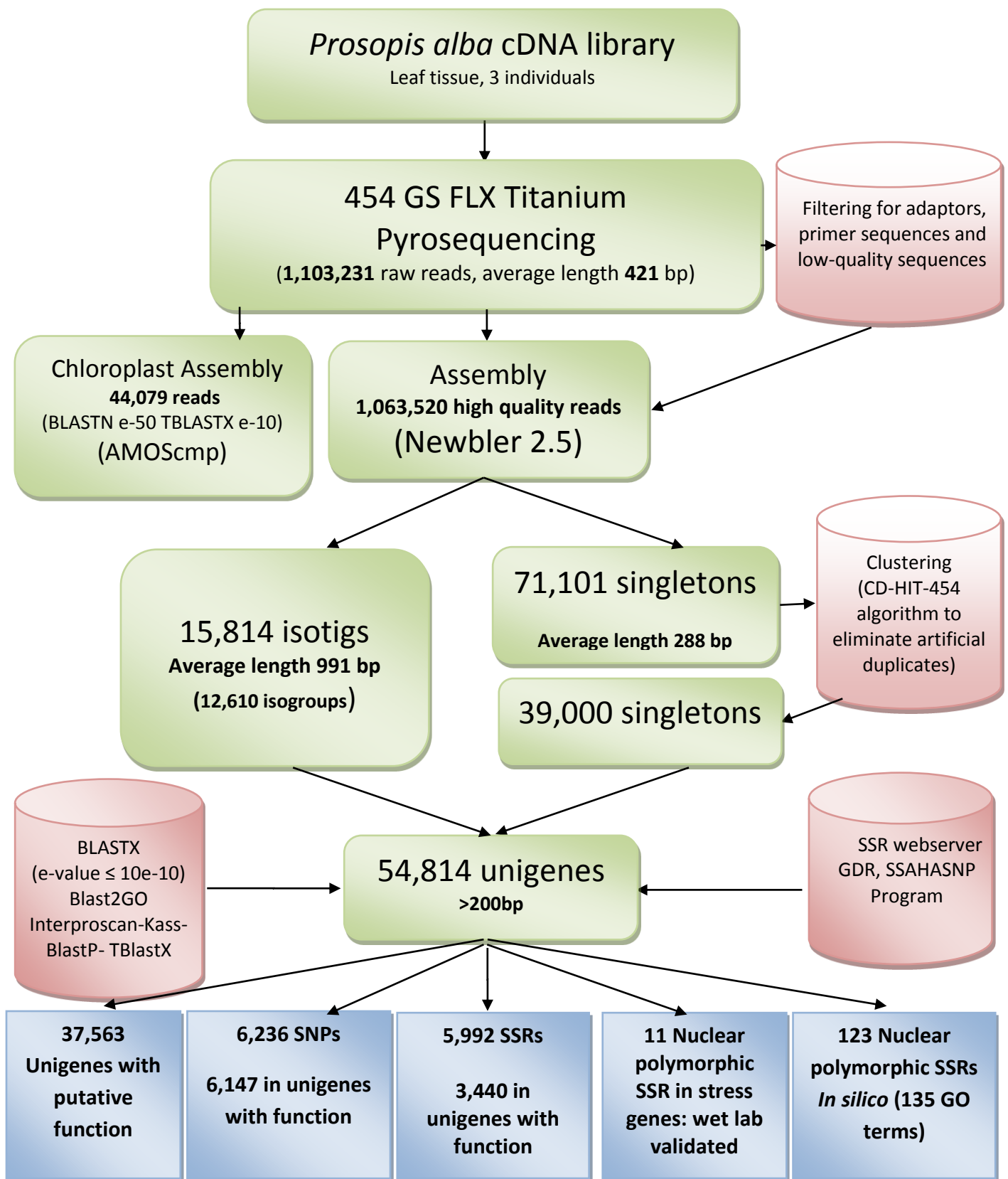

Supplement: Additional file 1 — Schematic representation of the overall sequencing and annotation workflow of Prosopis alba transcriptome. The steps and sets of sequences involved in transcriptome sequencing, assembly of reads, annotation using protein databases, the statistical thresholds, filters, genetic marker discovery and characterization. [file 1471-2164-14-705-S1.pdf]
